# Successful Simultaneous Subtotal Splenectomy During Left Lobe Auxiliary Liver Transplantation for Portal Inflow Modulation and Severe Hypersplenism Correction: A Case Report

**DOI:** 10.3389/fmed.2021.818825

**Published:** 2022-01-31

**Authors:** Guang-Peng Zhou, Wei Qu, Zhi-Gui Zeng, Li-Ying Sun, Ying Liu, Lin Wei, Zhi-Jun Zhu

**Affiliations:** ^1^Liver Transplantation Center, National Clinical Research Center for Digestive Diseases, Beijing Friendship Hospital, Capital Medical University, Beijing, China; ^2^Clinical Center for Pediatric Liver Transplantation, Capital Medical University, Beijing, China; ^3^Department of Critical Liver Diseases, Liver Research Center, Beijing Friendship Hospital, Capital Medical University, Beijing, China

**Keywords:** subtotal splenectomy, auxiliary liver transplantation, portal inflow modulation, hypersplenism, thrombocytopenia

## Abstract

Adult-to-adult living donor liver transplantation with small partial liver grafts often requires intraoperative portal inflow modulation to prevent portal hyperperfusion and subsequent small-for-size syndrome (SFSS). However, there are concerns about the specific morbidity of these modulation techniques. This study aims to lower post-perfusion portal venous pressure and correct severe hypersplenism in a patient with end-stage liver cirrhosis by simultaneous subtotal splenectomy during auxiliary partial orthotopic liver transplantation (APOLT). A 29-year-old man was diagnosed with cryptogenic cirrhosis and severe portal hypertension suffered recurrent acute variceal bleeding, severe thrombocytopenia, and massive ascites before admission to our hospital. After the recipient's left liver was resected, we performed APOLT using his 51-year-old father's left lobe graft with a graft-to-recipient weight ratio of 0.55%. Intraoperatively, simultaneous subtotal splenectomy was performed to lower graft post-perfusion portal vein pressure below 15 mmHg and correct severe hypersplenism-related pancytopenia. The recipient's postoperative hospital course was uneventful with no occurrence of SFSS and procedure-related complications. Platelet and leukocyte counts remained in the normal ranges postoperatively. The living donor was discharged 6 days after the operation and recovered well-with no complications. After a follow-up period of 35.3 months, both the recipient and donor live with good liver function and overall condition. This is the first case report of simultaneous subtotal splenectomy during APOLT using small-for-size living-donated left liver lobes, which is demonstrated to be a viable procedure for modulating portal inflow and correcting severe hypersplenism in selected adult patients with end-stage liver cirrhosis. APOLT using a small-for-size liver graft may be a safe and feasible treatment option for selected adult patients with end-stage liver cirrhosis.

## Introduction

Due to a critical shortage of donor organs, living donor liver transplantation (LDLT) has been increasingly used in adult recipients. In general, a graft ≥0.8% of the recipient's body weight or 30–40% of standard liver volume (SLV) is necessary to meet the recipient's functional demands, whereas procuring such a graft from a living donor may subject a healthy individual to a significantly high operative risk (1). To ensure donor safety and expand the donor pool, small-for-size grafts (SFSGs), defined as grafts with a graft-to-recipient weight ratio (GRWR) <0.8%, are being widely used in clinical practice. However, SFSGs are thought to be the leading cause of small-for-size syndrome (SFSS) associated with poorer prognosis (1, 2).

Auxiliary partial orthotopic liver transplantation (APOLT), which is implanting a normal partial liver allograft while preserving part of the native liver, has emerged as an attractive alternative to liver transplantation (LT) for the treatment of selected patients (3, 4). The implanted partial liver graft and residual partial native liver will co-function and support each other, which can balance the risk of donors and the need of recipients. In fulminant liver failure, the auxiliary liver graft is implanted to temporarily provide emergent support for the loss of native liver function, offering a chance to the potential spontaneous recovery of the native liver, and then allowing for possible progression to immunosuppression withdrawal (5, 6). In non-cirrhotic inherited metabolic liver disease, the implanted small graft can compensate for the recipient's enzyme deficiency without the complete removal of the native liver. The implanted partial liver graft and the residual non-cirrhotic native liver can permanently cooperate to maintain normal liver function (7, 8). In chronic cirrhotic liver disease, the residual native liver with relatively preserved liver function can temporarily support the implanted small volume graft during the immediate postoperative period until sufficient regeneration (3, 9–11). Recently, Brunner et al. reported two successful cases of auxiliary two-staged partial resection LT using living-donor left lobes (GRWR 0.65 and 0.43%, respectively) for end-stage liver cirrhosis to prevent SFSS (12). Together, APOLT using a small volume liver graft may be a feasible therapeutic option for adult patients with end-stage liver cirrhosis.

Post-transplant portal hyperperfusion is believed to be a primary contributor to the pathophysiology of SFSS. In this setting, intraoperative portal inflow modulation techniques, including splenectomy, splenic artery ligation, and portosystemic shunts, can be used to optimize the post-perfusion portal vein pressure (PVP) and thus prevent SFSS-related graft loss (13, 14). Yoshizumi et al. found that simultaneous total splenectomy should be recommended for patients with severe portal hypertension or high portal pressure (>20 mmHg) after reperfusion in adult LDLT (15). Thrombocytopenia in the immediate postoperative period after LDLT is a common phenomenon in patients with end-stage liver cirrhosis (16). Prolonged thrombocytopenia can expose liver transplant recipients to a high risk of severe and even life-threatening hemorrhagic complications, thereby complicating the postoperative clinical course and even resulting in increased morbidity and mortality (16, 17). A recent study by Pamecha et al. showed that postoperative severe thrombocytopenia was associated with a high risk of early graft dysfunction, prolonged ascites drainage, and sepsis in LDLT recipients (17). It has been previously reported that simultaneous splenectomy has a beneficial role in adult LDLT to correct hypersplenism-related pancytopenia (18). However, splenectomy-related lethal complications, including hemorrhage from the splenectomy bed, thromboembolic and septic complications, pancreatic fistula and/or abscess, might overshadow the benefits of splenectomy (15, 19, 20).

The present case describes an adult patient diagnosed with cryptogenic cirrhosis and severe portal hypertension who underwent APOLT using a small volume living-donated left liver lobe. Simultaneous subtotal splenectomy was performed intraoperatively to lower graft PVP and correct severe hypersplenism-related pancytopenia.

## Case Report

A 29-year-old man was admitted to our hospital because of recurrent variceal bleeding. One year ago, he presented with sudden melena and was diagnosed with cryptogenic cirrhosis and severe portal hypertension at the local hospital. Since then, he had experienced seven episodes of acute variceal bleeding despite multiple endoscopic therapies (including endoscopic injection sclerotherapy and endoscopic variceal ligation) and regular administration of non-selective beta-blockers (carvedilol). Two months ago, he suffered from a hemorrhagic shock caused by gastrointestinal bleeding. On this admission, he had melena and his blood routine examination revealed moderate anemia (hemoglobin 75 g/l) and severe hypersplenism (platelet count 13,000/mm^3^ and white blood cell count 600/mm^3^). Serum biochemical examinations showed slightly elevated total bilirubin (2.0 mg/dl) and conjugated bilirubin (0.57 mg/dl), normal aspartate aminotransferase (26.6 IU/l) and alanine aminotransferase (6 IU/l), normal serum creatinine (67 μmol/l) and electrolytes (sodium 139.8 mmol/l, potassium 4.16 mmol/l, chloride 112 mmol/l) and low albumin level (2.91 g/dl). The blood ammonia level was 33 μmol/l, and the patient did not experience any episodes of hepatic encephalopathy previously. His international normalized ratio (INR) was slightly prolonged (1.49). Abdominal computed tomography (CT) showed atrophic liver, severely dilated gastroesophageal varices and short gastric veins, umbilical vein recanalization, splenomegaly, massive volume of free peritoneal fluid, and small volume of bilateral pleural effusion. The Child–Pugh classification was B (score 9), and the Model for End-Stage Liver Disease (MELD) score was 12. After a discussion between the patient and his parents and the multidisciplinary team, including radiologists, hepatologists, and liver transplant surgeons, the patient refused to undergo partial splenic embolization, surgical shunt or transjugular intrahepatic portosystemic shunt because the patient thought that abovementioned procedures were just temporary therapeutic options but not a cure, with the potential high risk of procedure-related complications including portal vein thrombosis, substantial infectious risk, and occurrence of hepatic encephalopathy, which may adversely influence his quality of life and the subsequent chance of LT. On the other hand, the indication for LT in this patient was relatively clear, namely decompensated liver cirrhosis, including portal hypertension, recurrent variceal hemorrhage, splenomegaly, hypersplenism, and moderate to severe ascites. In such a situation, considering the patient's own wish regarding the LT and the donor's safety being the priority during LDLT, as well as our transplant team's previous extensive experience with APOLT, and thus APOLT using a small-for-size living-donated left liver lobe was considered for this patient.

**Figure 1:**
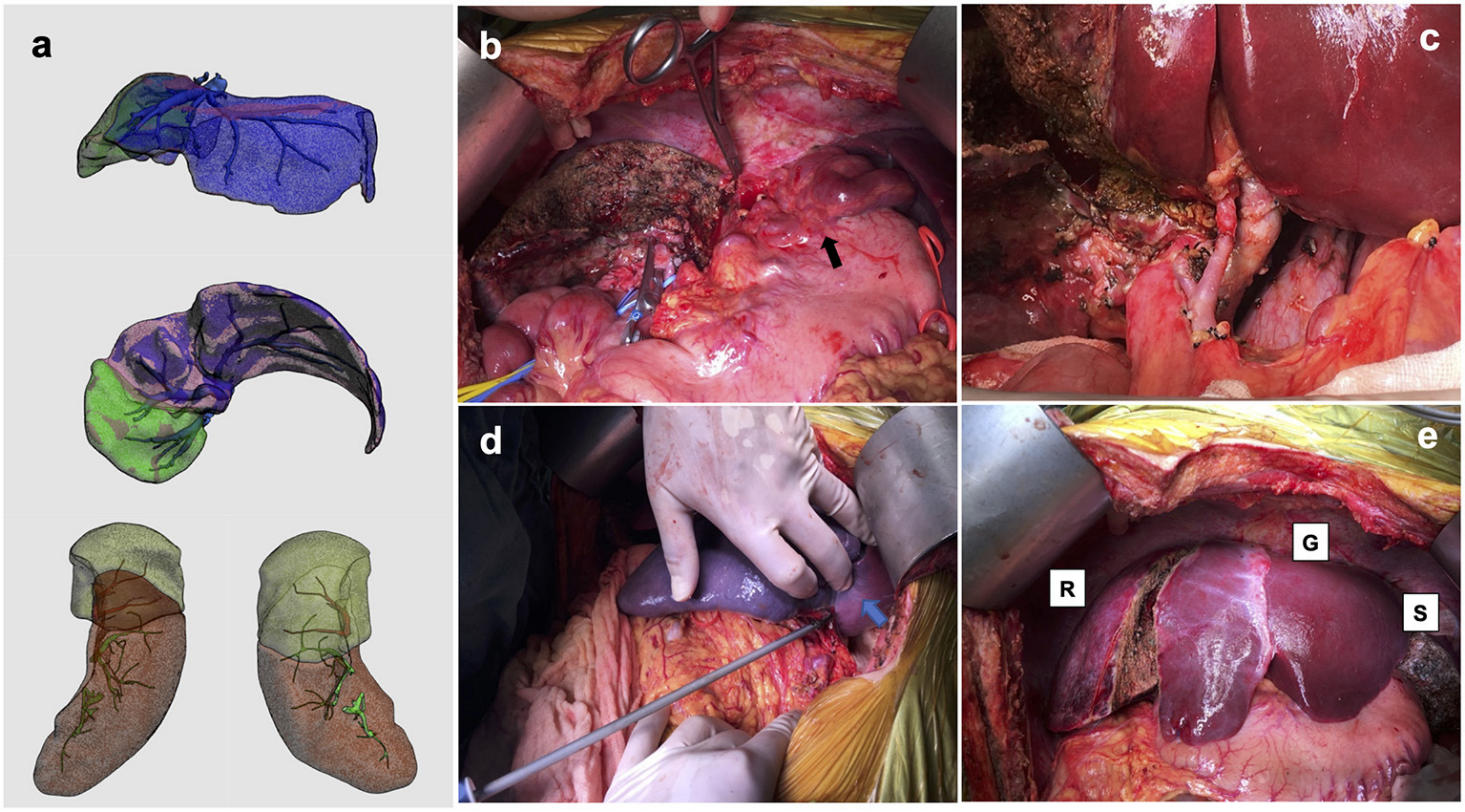
Estimated hepatic and splenic volumes of the recipient and photographs of the operating field. **(a)** Calculated hepatic volume was 905 ml (volume of planned preserved right hemi-liver was 270 ml), and splenic volume was 1,445 ml (volume of designed preserved upper pole was 503 ml; **(b)** remnant right hemi-liver and large varicose veins (black arrow); **(c)** hepatic artery and portal vein to auxiliary partial graft; **(d)** ischemic demarcation line on spleen (blue arrow) during simultaneous subtotal splenectomy; **(e)** native remnant liver (R), partial liver graft (G) and residual spleen (S).

The patient's 51-year-old father was evaluated to be a suitable living donor. Considering the advanced age of the donor, we planned procurement of the left lobe liver to ensure the donor's safety. Using the three-dimensional imaging reconstruction analysis system, the estimated left lobe graft volume was 476 ml, representing an estimated GRWR of 0.65%, and graft volume (GV)/SLV was 36.71%. Thus, APOLT using a left lobe liver graft was designed to decrease the morbidity of SFSS in the recipient. Given the presence of severe portal hypertension, hypersplenism, and massive splenomegaly, simultaneous subtotal splenectomy was planned, which served as a portal inflow modulation strategy and treatment for severe hypersplenism. The calculated volume of the whole spleen and the designed preserved upper pole of the spleen were 1,445 and 503 ml, respectively (Figure 1a).

The donor underwent a left hemihepatectomy with preservation of the middle hepatic vein, and the intraoperative course was uncomplicated. The actual graft weight was 405 g, GRWR was 0.55%, and GV/SLV was 31.23%. The recipient underwent APOLT with preservation of the native right lobe liver (Figure 1b). Before hepatectomy, PVP was measured with a needle inserted directly in the portal vein trunk; the value of which was 26 mmHg. The graft was implanted using a modified piggyback technique with an end-to-end anastomosis between the left hepatic vein of the donor graft and the common left/middle hepatic vein orifice of the recipient. The end-to-end portal anastomosis was performed between the graft's left portal vein and the recipient's left portal vein stump. The graft's left hepatic artery was anastomosed to the recipient's left hepatic artery (Figure 1c). Graft PVP measurement was 19 mmHg after reperfusion and decreased to 12 mmHg upon temporary clamping of the splenic artery trunk. Subtotal splenectomy was performed by ligating the corresponding segmental arteries and veins of the lower two-thirds of the spleen, as previously described (21). About 70% of the splenic volume was resected (Figure 1d), and the final graft PVP was 14 mmHg. Biliary anastomosis was performed between the graft left bile duct and the recipient left bile duct (Figure 1e). The warm and cold ischemia time of the graft was 23 and 276 min, respectively. The total operating time was 770 min (the time of subtotal splenectomy was about 39 min). Intraoperatively, the patient remained hemodynamically stable without unexpected complications. The intraoperative blood loss was ~750 ml.

**Figure 2:**
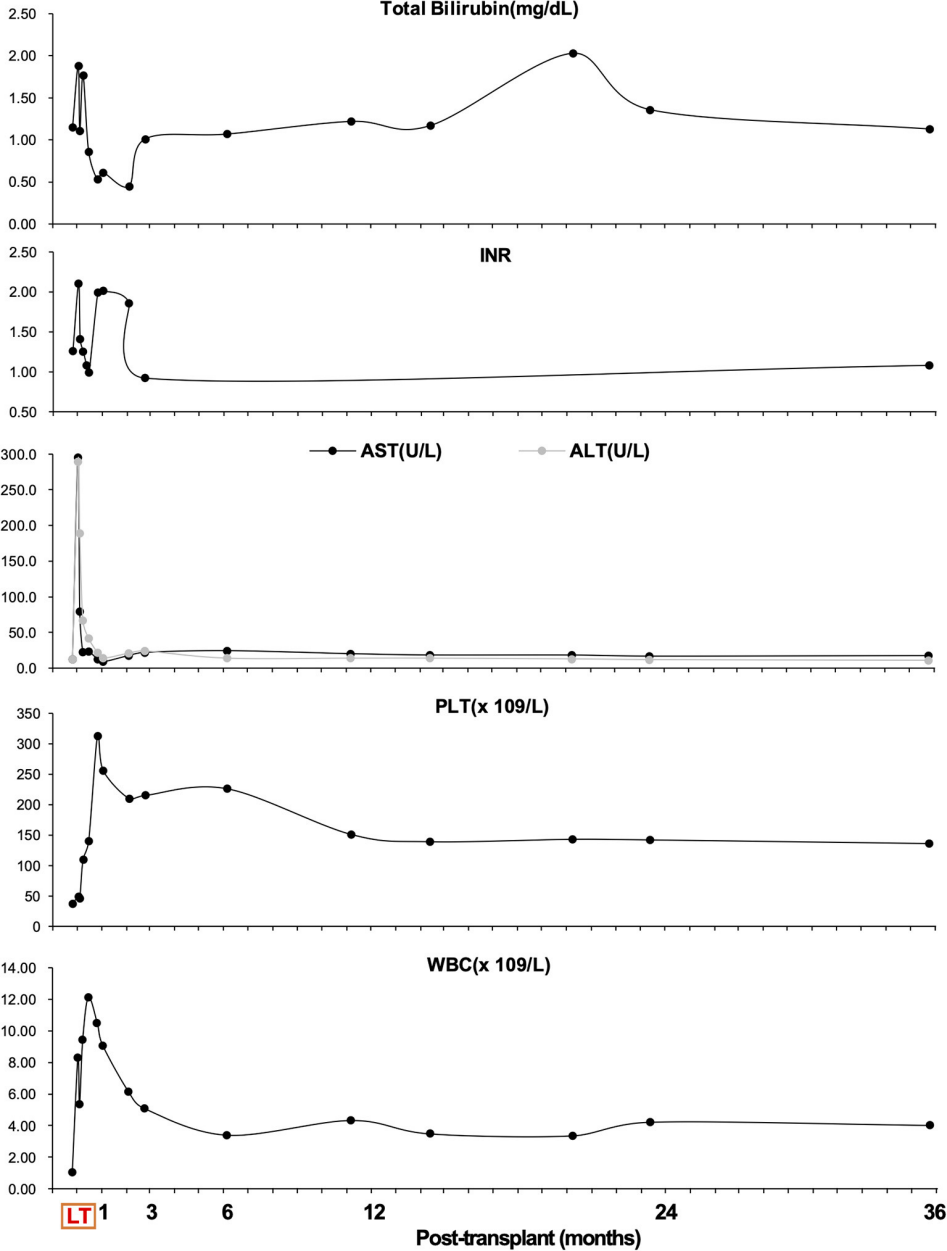
Overview of total bilirubin (mg/dl), international normalized ratio (INR), aspartate aminotransferase (AST, U/L), alanine aminotransferase (ALT, U/L), platelet (PLT, 10^9^/L) count, and white blood cell (WBC, 10^9^/L) count during follow-up.

Based on our and other centers' experience, vaccination against pneumococci and any other encapsulated bacteria was not performed before or after transplantation (18, 21). To prevent vascular thrombosis, unfractionated heparin sodium (10,000 U/day) was started from the postoperative day (POD) 3–7. Then anticoagulation using warfarin was added to maintain INR 1.5–2.0 until postoperative month 3 (Figure 2). Postoperative abdominal Doppler ultrasonography was performed to examine flow in the graft vessels at least once daily until POD 7 and once every 2 days between POD 8 and 14. Abdominal dynamic contrast-enhanced CT was performed if ultrasound abnormalities were noticed. Postoperative immunosuppressive therapy consisted of methylprednisolone, mycophenolate mofetil, and tacrolimus. Pathological analysis of the excised liver specimen suggested cirrhosis.

**Figure 3:**
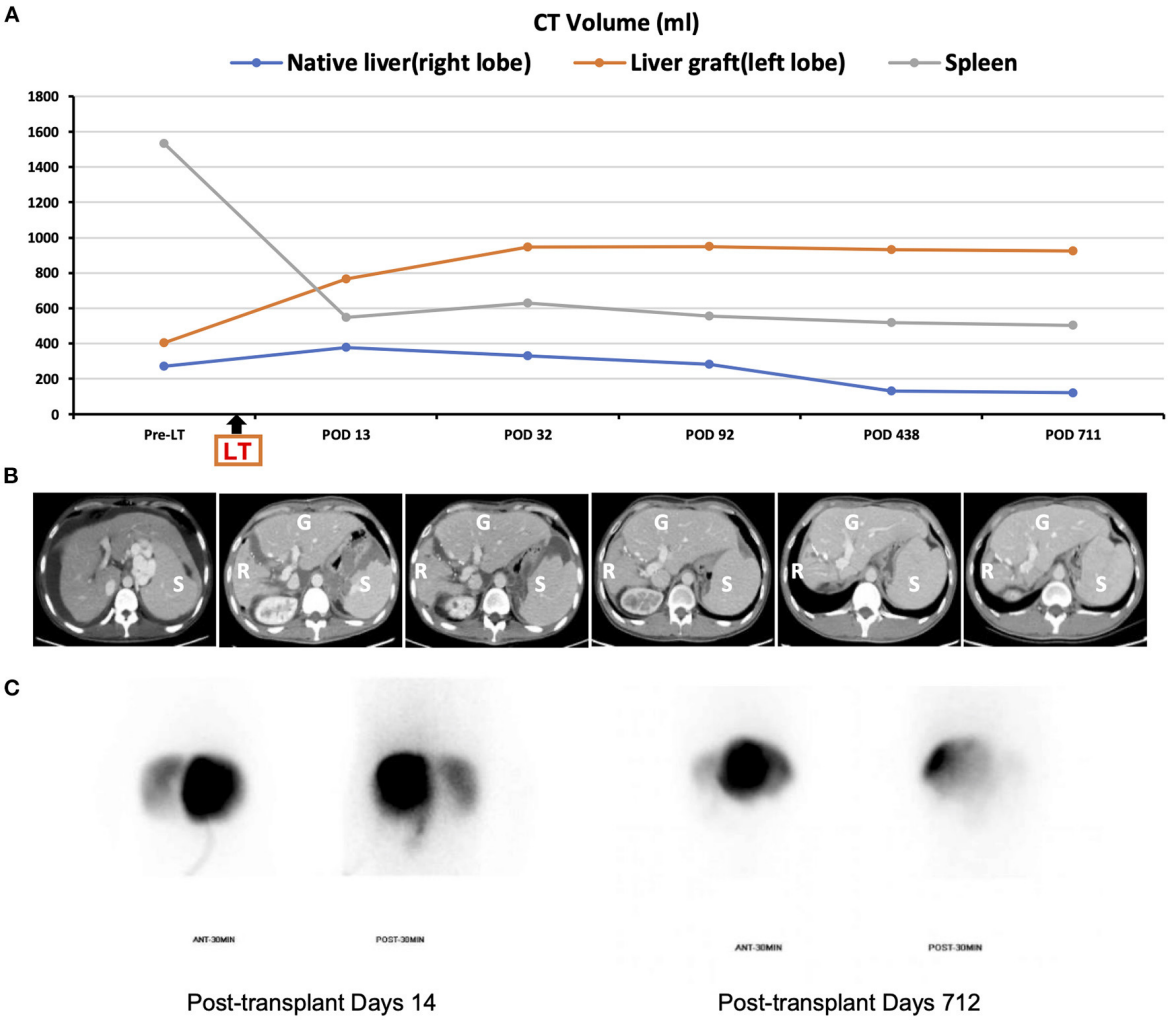
Pre- and postoperative imaging. **(A)** Changes in CT-simulated volume of the native residual liver, graft, and spleen throughout follow-up; **(B)** representative abdominal CT images (R, liver remnant; G, graft; S, spleen); **(C)** Tc-99 m hepatobiliary scintigraphy on post-transplant day 14 and 712 (left liver graft: right native liver = 88%: 12 and 97.9%: 2.1%, respectively).

Apart from massive ascites production, the postoperative hospital course was uneventful, and graft function recovered rapidly (Figure 2). It took 6 weeks to achieve disappearance of abdominal drainage, and then the drainage tube was removed. The platelet and leukocyte counts increased immediately during the perioperative period and remained in the normal ranges during long-term follow-up (Figure 2). There was no occurrence of splenectomy-related complications such as bleeding from splenectomy bed, portal venous thrombosis, pancreatic fistula and abscess, or septic complications. The recipient was discharged from the hospital on day 43 post-transplant. He was followed up regularly according to his clinical and laboratory data. The anatomical and functional volume of the implanted liver graft and residual native liver were evaluated using a dynamic contrast-enhanced CT scan and 99 mTc-GSA scintigraphy. The results showed gradually increased volume and enhanced function of the liver graft (Figure 3). The volume of the remnant spleen was also monitored, indicating no occurrence of splenic regrowth (Figure 3A). Moreover, there were no procedure-related complications throughout the follow-up period. To date (35.3 months post-transplant), the patient's graft function is good, and peripheral platelet and leukocyte counts remain in the normal ranges (Figure 2). Meanwhile, the living donor recovered well-with no postoperative complications. He was discharged in good condition 6 days after the operation, and the clinical course after discharge was uneventful. This study was conducted in accordance with the Declaration of Helsinki. It was approved by the Ethical Committee of Beijing Friendship Hospital, Capital Medical University (No. 2018-P2-127-01). The patient has signed informed consent.

## Discussion

With a critical shortage of donated organs and increased mortality for those on the waiting list, living donors have become an increasingly accepted potential alternative source of organs for adult patients with end-stage liver disease. Due to the assurance of donor's safety being the priority in LDLT, SFSGs have been used to minimize risks to donors in adult-to-adult LDLT. However, SFSGs are generally considered unsafe in terms of the risk of SFSS (22). Notably, the occurrence of SFSS is not only attributed to graft size mismatch but also likely to be associated with other factors, including graft quality (donor age and steatosis), recipient conditions (portal hypertension and severity of original liver disease), and technical issues (venous reconstruction) (23, 24). In the present study, insufficient graft parenchyma, advanced donor age, and severe portal hypertension may have induced excessive portal blood flow through the small-volume graft, leading to a high risk of postoperative SFSS and early allograft dysfunction. APOLT, which was initially explored for potentially reversible fulminant liver failure (temporary support by the auxiliary graft), has been described in patients with liver-based metabolic diseases, chronic liver disease and small liver grafts (temporary or permanent support by the native liver remnant) (3, 4, 8, 10). Therefore, APOLT with the preservation of the right lobe, which can lower the volume requirement for the graft, was performed in our recipient. It was anticipated that the residual native liver with relatively preserved liver function could temporarily support the function of the implanted small-for-size left liver lobe during the immediate postoperative period until the small graft had regenerated sufficiently. Then the graft can be expected to fully meet the hepatic functional demands of the recipient. More recently, a similar approach known as auxiliary two-staged partial resection LT using living-donor left lobes has been reported as a safe and technically feasible treatment for end-stage liver disease to avoid the small-for-size situation in adults/adolescents (12). Here, our case further demonstrated the clinical feasibility of APOLT using small-for-size living-donated left liver lobes for selected adult patients with end-stage liver cirrhosis, allowing increasing the pool of liver grafts and decreasing the risk of SFSS.

Notably, the functional competition of the portal vein flow between the remnant native liver and the implanted graft is a significant concern of APOLT, leading to progressive growth of one-side conquering liver and chronic atrophy of the defeated liver (4, 25). In our patient, due to the pre-existence of cirrhosis contributing to consequent higher intrahepatic vascular resistance in the remnant native liver, the portal blood flow to the soft implanted graft had an advantage over that to the cirrhotic residual liver. By virtue of the superiority of portal vein blood flow, the small liver graft was expected to expand its function in proportion to volume growth until it met the hepatic functional demand of the recipient with sufficient volume, while the native liver would shrink gradually. In fact, the results of postoperative abdominal dynamic contrast-enhanced CT scan and 99 mTc-GSA scintigraphy showed a progressive increase in the volume of the implanted liver graft and a gradual decrease in that of the residual native liver (Figure 3). Due to the inherent benign nature of the disease, namely cryptogenic cirrhosis, issues of disease transmission from the remnant native liver to graft can be ignored; therefore, removing the native cirrhotic liver was not considered in this recipient. Notably, however, given the potential risk of carcinogenicity of the remnant native liver, the patient underwent a hepatocellular carcinoma surveillance program including abdominal ultrasound, serum alpha-fetoprotein and prothrombin induced by vitamin K absence-II during his regular follow-up.

Previous studies have suggested that preoperative graft-to-spleen volume ratio (GSVR) at a ratio of <0.6 g/ml is a good predictor of the development of post-transplant portal hyperperfusion; therefore, it can be used to indicate whether splenectomy is required before reperfusion (26, 27). Yao et al. reported that PVP >15 mmHg was associated with poor prognosis in grafts from donors aged ≥45 years, and lowering PVP to ≤ 15 mmHg was a key to prevention of SFSS and consequent early graft loss (28). Given the high PVP after reperfusion and advanced donor age, intraoperative portal inflow modulation was necessary for our recipient. Currently, there are several available strategies for portal inflow modulation, including splenic artery ligation, splenectomy, and portosystemic shunts, while concerns about these procedures remain (29, 30). Splenic artery ligation has limited effect on PVP control and carries a risk of sepsis and insufficient recovery of pancytopenia (29, 31); simultaneous splenectomy could be associated with the risk of portal hypoperfusion, portal venous thrombosis, infectious complications, and postoperative bleeding (20, 32); portosystemic shunts carry a risk of the development of portal steal phenomenon, causing excessive diversion of the portal flow to the systemic circulation (27, 31).

More recently, Zhou and Wei et al. performed simultaneous partial splenectomy during LT in patients with severe hypersplenism, which achieved a satisfactory long-term hematological response but avoided untoward complications of total splenectomy (21, 33). A recent study by Yao et al. found that an extremely low GSVR ( ≤ 0.7 g/ml) was associated with persistent hypersplenism, impaired graft function, and consequent early graft loss after LDLT in patients with spleen preservation (34). Furthermore, it has been proved that preoperative spleen volume and low platelet count may contribute to post-transplant persistent thrombocytopenia, complicating the postoperative course among liver transplant recipients (16, 35, 36). Given our recipient's high graft PVP (19 mmHg) after reperfusion, large spleen (GSVR = 0.28 g/ml) and severe hypersplenism, we performed subtotal splenectomy at the time of transplantation, aiming to decompress graft portal hyperperfusion and correct hypersplenism-related pancytopenia. Apart from a long period of abdominal drainage, our recipient showed no noticeable signs of post-reperfusion portal hypertension and consequent complications in the early postoperative period. Platelet and leukocyte counts remained in the normal ranges throughout follow-up, suggesting severe pancytopenia related to hypersplenism had also been completely corrected.

## Conclusion

This case suggests that simultaneous subtotal splenectomy during APOLT using small-for-size living-donated left liver lobes is not only an efficient intraoperative portal inflow modulation strategy but also a feasible treatment for severe hypersplenism in selected adult patients with end-stage liver cirrhosis. It could be a viable alternative to splenectomy, which preserves splenectomy's beneficial effects but avoids splenectomy-related lethal complications. Furthermore, APOLT using a small-for-size liver graft may be a safe and feasible therapeutic option for selected adult patients with end-stage liver cirrhosis, which can further expand the pool of donor grafts.

## Data Availability Statement

The raw data supporting the conclusions of this article will be made available by the authors, without undue reservation.

## Ethics Statement

The studies involving human participants were reviewed and approved by the Ethical Committee of Beijing Friendship Hospital, Capital Medical University (No. 2018-P2-127-01). The patients/participants provided their written informed consent to participate in this study and for the publication of this case report.

## Author Contributions

Z-JZ and LW: conception and design of the research, analysis and interpretation of the data, and preformation of the operations. G-PZ: acquisition of data, statistical analysis, and writing of the initial draft of manuscript. WQ, Z-GZ, YL, and L-YS: clinical management and follow-up of the patient. All authors involved in the critical revision of the manuscript for important intellectual content and approved the final version of the manuscript.

## Funding

The present work was supported by the Capital's Funds for Health Improvement and Research (Grant/Award Number: 2020-1-2024) and Beijing Municipal Science and Technology Commission (No. Z211100002921026). The funding body had no role in the design of the study and collection, analysis, and interpretation of data, and in writing the manuscript.

## Conflict of Interest

The authors declare that the research was conducted in the absence of any commercial or financial relationships that could be construed as a potential conflict of interest.

## Publisher's Note

All claims expressed in this article are solely those of the authors and do not necessarily represent those of their affiliated organizations, or those of the publisher, the editors and the reviewers. Any product that may be evaluated in this article, or claim that may be made by its manufacturer, is not guaranteed or endorsed by the publisher.

## Abbreviations

SFSS: small-for-size syndrome
APOLT: auxiliary partial orthotopic liver transplantation
LDLT: living donor liver transplantation
LT: liver transplantation
SLV: standard liver volume
SFSG: small-for-size graft
GRWR: graft-to-recipient weight ratio
PVP: portal vein pressure
GV: graft volume
POD: postoperative day
INR: international normalized ratio
CT: computed tomography
MELD: Model for End-Stage Liver Disease
GSVR: graft-to-spleen volume ratio.

## References

[1] Dahm F Georgiev P Clavien PA. Small-for-size syndrome after partial liver transplantation: definition, mechanisms of disease and clinical implications. Am J Transpl. (2005) 5:2605–10. 10.1111/j.1600-6143.2005.01081.x 16212618

[2] Yoichi T Takayashiki T Shimizu H Yoshidome H Ohtsuka M Kato A. Protective effects of simultaneous splenectomy on small-for-size liver graft injury in rat liver transplantation. Transpl Int. (2014) 27:106–13. 10.1111/tri.12223 24164377

[3] Wang SF Chen XP Chen ZS Wei L Dong SL Guo H. Left lobe auxiliary liver transplantation for end-stage hepatitis b liver cirrhosis. Am J Transpl. (2017) 17:1606–12. 10.1111/ajt.14143 27888553

[4] Kasahara M Takada Y Egawa H Fujimoto Y Ogura Y Ogawa K. Auxiliary partial orthotopic living donor liver transplantation: Kyoto University experience. Am J Transpl. (2005) 5:558–65. 10.1111/j.1600-6143.2005.00717.x 15707411

[5] Quadros J Piedade C Lopes MF. Auxiliary liver transplantation for management of acute liver failure in children - Systematic review. Transpl Rev. (2021) 35:100631. 10.1016/j.trre.2021.100631 34098491

[6] Rela M Kaliamoorthy I Reddy MS. Current status of auxiliary partial orthotopic liver transplantation for acute liver failure. Liver Transpl. (2016) 22:1265–74. 10.1002/lt.24509 27357489

[7] Kasahara M Sakamoto S Horikawa R Fukuda A. Auxiliary partial orthotopic liver transplantation for noncirrhotic metabolic liver disease: reigniting interest in an old but new technique. Liver Transpl. (2019) 25:12–3. 10.1002/lt.25388 30472792

[8] Qu W Wei L Zhu ZJ Sun LY Liu Y Zeng ZG. Considerations for use of domino cross-auxiliary liver transplantation in metabolic liver diseases: a review of case studies. Transplantation. (2019) 103:1916–20. 10.1097/tp.0000000000002602 30801517

[9] Wei L Zhang HM Wan CD Qu W Zeng ZG Liu Y. Auxiliary liver graft can be protected from HBV infection in hbsag positive blood circulation. Front Med. (2021) 8:726502. 10.3389/fmed.2021.726502 34513885 PMC8423919

[10] Dokmak S Elkrief L Belghiti J. Auxiliary liver transplantation with a small deceased liver graft for cirrhotic liver complicated by hepatocellular carcinoma. Transpl Int. (2013) 26:e102–4. 10.1111/tri.12173 23964699

[11] Kasahara M Kiuchi T Uryuhara K Takakura K Egawa H Asonuma K. Auxiliary partial orthotopic liver transplantation as a rescue for small-for-size grafts harvested from living donors. Transpl Proc. (1998) 30:132–3. 10.1016/s0041-1345(97)01210-4 9474980

[12] Brunner SM Brennfleck FW Junger H Grosse J Knoppke B Geissler EK. Successful auxiliary two-staged partial resection liver transplantation (ASPIRE-LTx) for end-stage liver disease to avoid small-for-size situations. BMC Surg. (2021) 21:166. 10.1186/s12893-021-01167-6 33771158 PMC7995706

[13] Ohno Y Mita A Ikegami T Masuda Y Urata K Nakazawa Y. Temporary auxiliary partial orthotopic liver transplantation using a small graft for familial amyloid polyneuropathy. Am J Transpl. (2012) 12:2211–9. 10.1111/j.1600-6143.2012.04061.x 22500969

[14] Singh Soin A Yadav SK Saha SK Rastogi A Bhangui P Thiagarajan S. Is portal inflow modulation always necessary for successful utilization of small volume live donor liver grafts? Liver Transpl. (2019) 25:1811–21. 10.1002/lt.25629 31436885

[15] Yoshizumi T Itoh S Shimokawa M Inokuchi S Harada N Takeishi K. Simultaneous splenectomy improves outcomes after adult living donor liver transplantation. J Hepatol. (2021) 74:372–9. 10.1016/j.jhep.2020.08.017 32827564

[16] Takahashi K Nagai S Safwan M Liang C Ohkohchi N. Thrombocytopenia after liver transplantation: should we care? World J Gastroenterol. (2018) 24:1386–97. 10.3748/wjg.v24.i13.1386 29632420 PMC5889819

[17] Pamecha V Mahansaria SS Kumar S Bharathy KG Sasturkar SV Sinha PK. Association of thrombocytopenia with outcome following adult living donor liver transplantation. Transpl Int. (2016) 29:1126–35. 10.1111/tri.12819 27429066

[18] Moon DB Lee SG Hwang S Ahn CS Kim KH Ha TY. Splenic devascularization can replace splenectomy during adult living donor liver transplantation - a historical cohort study. Transpl Int. (2019) 32:535–45. 10.1111/tri.13405 30714245

[19] Ito K Akamatsu N Ichida A Ito D Kaneko J Arita J. Splenectomy is not indicated in living donor liver transplantation. Liver Transpl. (2016) 22:1526–35. 10.1002/lt.24489 27253521

[20] Kurata N Ogura Y Ogiso S Onishi Y Kamei H Kodera Y. Splenectomy in living donor liver transplantation and risk factors of portal vein thrombosis. Hepatobil Pancreat Dis Int. (2019) 18:337–42. 10.1016/j.hbpd.2019.06.011 31278029

[21] Wei L Zhou GP Qu W Zeng ZG Sun LY Liu Y. Is simultaneous partial splenectomy during pediatric liver transplantation safe and effective for severe hypersplenism? A prospective cohort study. Int J Surg. (2021) 88:105926. 10.1016/j.ijsu.2021.105926 33746054

[22] Line PD Hagness M Berstad AE Foss A Dueland S. A novel concept for partial liver transplantation in nonresectable colorectal liver metastases: the rapid concept. Ann Surg. (2015) 262:e5–9. 10.1097/SLA.0000000000001165 25692361

[23] Raut V Alikhanov R Belghiti J Uemoto S. Review of the surgical approach to prevent small-for-size syndrome in recipients after left lobe adult LDLT. Surg Today. (2014) 44:1189–96. 10.1007/s00595-013-0658-6 23904045

[24] Macshut M Kaido T Yao S Yagi S Ito T Kamo N. Older donor age is a risk factor for negative outcomes after adult living donor liver transplantation using small-for-size grafts. Liver Transpl. (2019) 25:1524–32. 10.1002/lt.25601 31298473

[25] Ikegami T Shimada M Imura S Arakawa Y Nii A Morine Y. Current concept of small-for-size grafts in living donor liver transplantation. Surg Today. (2008) 38:971–82. 10.1007/s00595-008-3771-1 18958553

[26] Cheng YF Huang TL Chen TY Concejero A Tsang LL Wang CC. Liver graft-to-recipient spleen size ratio as a novel predictor of portal hyperperfusion syndrome in living donor liver transplantation. Am J Transpl. (2006) 6:2994–9. 10.1111/j.1600-6143.2006.01562.x 17061990

[27] Gyoten K Mizuno S Kato H Murata Y Tanemura A Azumi Y. A novel predictor of posttransplant portal hypertension in adult-to-adult living donor liver transplantation: increased estimated spleen/graft volume ratio. Transplantation. (2016) 100:2138–45. 10.1097/TP.0000000000001370 27472097 PMC5120765

[28] Yao S Kaido T Uozumi R Yagi S Miyachi Y Fukumitsu K. Is portal venous pressure modulation still indicated for all recipients in living donor liver transplantation? Liver Transpl. (2018) 24:1578–88. 10.1002/lt.25180 29710397

[29] Hernandez-Alejandro R Sharma H. Small-for-size syndrome in liver transplantation: new horizons to cover with a good launchpad. Liver Transpl. (2016) 22:33–6. 10.1002/lt.24513 27398648

[30] Ikegami T Balci D Jung DH Kim JM Quintini C. Living donor liver transplantation in small-for-size setting. Int J Surg. (2020) 82s:134–7. 10.1016/j.ijsu.2020.07.003 32738547

[31] Wang H Ikegami T Harada N Yoshizumi T Soejima Y Uchiyama H. Optimal changes in portal hemodynamics induced by splenectomy during living donor liver transplantation. Surg Today. (2015) 45:979–85. 10.1007/s00595-014-0999-9 25080864

[32] Badawy A Hamaguchi Y Satoru S Kaido T Okajima H Uemoto S. Evaluation of safety of concomitant splenectomy in living donor liver transplantation: a retrospective study. Transpl Int. (2017) 30:914–23. 10.1111/tri.12985 28512755

[33] Zhou GP Wei L Zhu ZJ Qu W Zeng ZG Sun LY. Successful simultaneous anatomic subtotal splenectomy during pediatric living-donor liver transplantation: a case report. Transpl Proc. (2020) 52:2767–72. 10.1016/j.transproceed.2020.01.170 32414607

[34] Yao S Kaido T Yagi S Uozumi R Iwamura S Miyachi Y. Impact of imbalanced graft-to-spleen volume ratio on outcomes following living donor liver transplantation in an era when simultaneous splenectomy is not typically indicated. Am J Transpl. (2019) 19:2783–94. 10.1111/ajt.15337 30830721

[35] Chen TY Chen CL Huang TL Tsang LL Ou HY Yu CY. Predictive factors for persistent splenomegaly and hypersplenism after adult living donor liver transplantation. Transpl Proc. (2012) 44:752–4. 10.1016/j.transproceed.2012.01.044 22483486

[36] Stanca CM Fiel MI Aledort L Cohen E Del Rio Martin J Schiano TD. Factors associated with persistent thrombocytopenia after liver transplantation. Transpl Proc. (2010) 42:1769–73. 10.1016/j.transproceed.2010.02.075 20620520

